# An α-chain modification rivals the effect of fetal hemoglobin in retarding the rate of sickle cell fiber formation

**DOI:** 10.1038/s41598-023-48919-3

**Published:** 2023-12-11

**Authors:** Eli H. Worth, Mark K. Fugate, Kimberly C. Grasty, Patrick J. Loll, Marilyn F. Bishop, Frank A. Ferrone

**Affiliations:** 1 Department of Physics, Drexel University, Philadelphia, PA 19104 USA; 2 Department of Biochemistry and Molecular Biology, Drexel University, Philadelphia, PA 19102 USA; 3 Deparment of Physics, Virginia Commonwealth University, Richmond, VA 23284-2000 USA

**Keywords:** Molecular medicine, Kinetics, Sickle cell disease

## Abstract

Adults with sickle cell disease bear a mutation in the β-globin gene, leading to the expression of sickle hemoglobin (HbS; α_2_β^S^_2_). Adults also possess the gene for γ-globin, which is a component of fetal hemoglobin (HbF, α_2_γ_2_); however, γ-chain expression normally ceases after birth. As HbF does not form the fibers that cause the disease, pharmacological and gene-modifying interventions have attempted to either reactivate expression of the γ chain or introduce a gene encoding a modified β chain having γ-like character. Here, we show that a single-site modification on the α chain, αPro114Arg, retards fiber formation as effectively as HbF. Because this addition to the repertoire of anti-sickling approaches acts independently of other modifications, it could be coupled with other therapies to significantly enhance their effectiveness.

## Introduction

Sickle cell disease results from the assembly of sickle hemoglobin (HbS) molecules into macroscopic fibers that compromise the flexibility of erythrocytes. Because the disease is caused by a single-nucleotide mutation in the β-globin gene, its cure by gene therapy has long been a dream that is now on the threshold of being realized^1,2^. Fortunately, complete replacement of the extant HbS is not required to provide substantial benefit. Instead, additional non-sickling hemoglobin variants can be introduced, with the goal of diluting the HbS and reducing fiber formation. In understanding the therapeutic potential of different hemoglobin variants, it is essential to recognize that all α_2_β_2_ hemoglobin tetramers exist in equilibrium with αβ dimers^3^. Hence, a mixture of HbS and any variant HbX will contain three types of tetramers: HbS, HbX, and HbS/HbX hybrids. The therapeutic effect will be greatest when neither the hybrid nor the HbX tetramers can add to the fibers. Such exclusion occurs for the HbF (α_2_γ_2_) variant, but does not occur in mixtures of HbS and wild-type (HbA) hemoglobin, for which the hybrid tetramers enter the sickle fiber^3^. Hence, the obvious gene therapy target of reintroducing normal β chains is not favored. An alternate approach is to introduce γ-chain genes into all cells^4^. Since the principal amino acid inhibiting fiber formation by γ-chain hybrids is thought to be γGln87, a β-chain variant has also been created containing the single mutation T87Q, and has shown promise in reducing disease pathology^5,6^. A variant using T87Q plus two additional β modifications is also in clinical trials (NCT02247843). Here we examine inhibiting fiber formation using a mutant on the α chain, HbChiapas (αP114R). While this mutant has been suggested as an anti-sickling variant^7^, the striking details of its kinetics were not previously known. We now show that it rivals HbF in inhibiting fiber formation.

The most appropriate metric for assessing the effectiveness of a hemoglobin variant is the rate of fiber formation, which determines the pathophysiology of sickle cell disease^8^. Fiber formation entails two nucleation-controlled pathways, both of which are always present and each of which has clinical implications^9^. In primary (homogeneous) nucleation, molecules in solution coalesce to form a nucleus from which a fiber will grow. Heterogeneous (secondary) nucleation accelerates fiber formation by allowing new fibers to nucleate on the surface of an existing fiber. Both pathways have physiological roles: If all fiber formation within a cell stems from a single primary nucleation event, the random variability of that event dominates the kinetics; whereas when multiple primary nuclei form, the kinetics of the secondary nucleation process control how rapidly a cell fills with fibers^9^.

We measured both nucleation processes by a continuous photolysis method^10^ wherein O_2_ is replaced as the heme ligand by CO, which is readily photodissociable from the heme. This allows us to use light to initiate and indefinitely sustain polymerization in a fully reversible fashion. We performed these experiments on mixtures of HbS and HbS Chiapas (i.e., HbS containing the additional αP114R mutation), and compared them with the rates of HbS/HbF mixtures.

**Figure 1:**
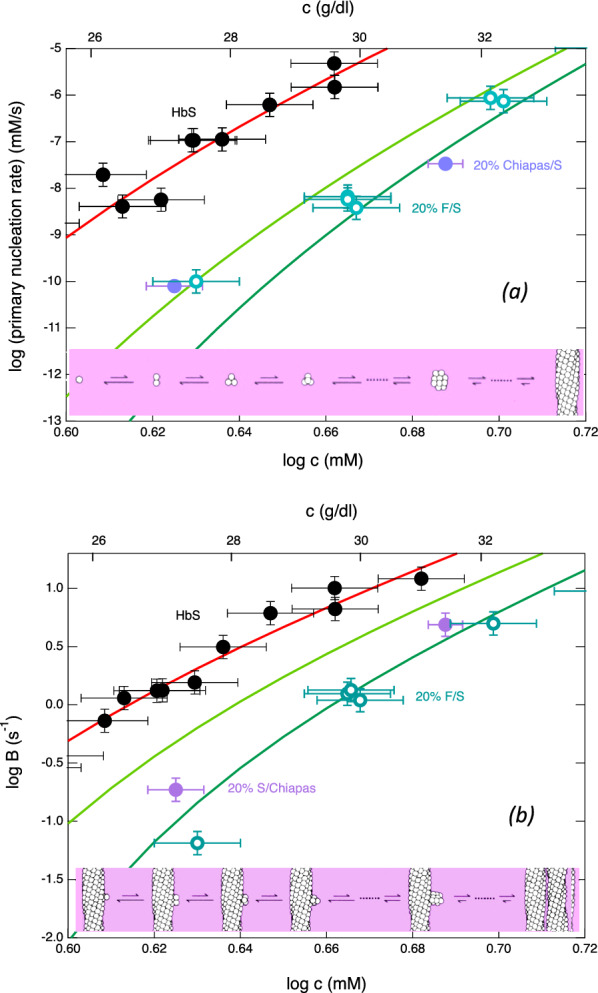
Nucleation kinetics as a function of total Hb concentration. (**a**) Primary (homogeneous) nucleation rate. The top (solid black) points show pure HbS^29–31^, while the open turquoise circles show 20% HbF mixed with 80% HbS^29^. The filled violet points show 20% HbS Chiapas mixed with 80% HbS. Vertical error bars show the uncertainty to the fits of the distributions, which typically involve over 100 points and yield the rate. Horizontal error bars result from measuring small volumes. The solid red line shows the theory developed to describe nucleation^9^; the lowest (dark green) line shows that theory adjusted for no copolymerization of hybrids (i.e., the effect of dilution alone)^29^. The middle line (light green) shows the theory if the hybrids have a probability of 10% to join the nucleus. (**b**) Exponential growth rate as a function of total Hb concentration. Secondary nucleation leads to exponential growth, so that the observed exponential factor (B) is dominated by that heterogeneous process. The symbols and lines are as in panel (**a**), except that the vertical error bars come from the fits to the exponential. Because the exponential factor B is related to the square root of the nucleation rate, its scale is smaller than the graph in panel (**a**).

Figure 1a shows the primary nucleation rate as a function of initial concentration. This reaction displays a remarkable concentration dependence (~ 57th order in the range shown). In mixtures of HbS with either 20% HbF or 20% HbS Chiapas, primary nucleation is suppressed roughly 1000-fold, as evidenced by a downward shift of the nucleation rate curve; importantly, HbS Chiapas suppresses as effectively as HbF. Figure 1b shows the effects of different Hb mixtures on secondary nucleation. Formation of secondary nuclei gives the reaction an exponential character, with the polymer mass growing as *Ae*^*Bt*^, where the growth parameter *B* is controlled by the secondary process^9^. As seen with primary nucleation, addition of HbF or HbS Chiapas significantly reduces *B* and thus secondary nucleation.

**Figure 2:**
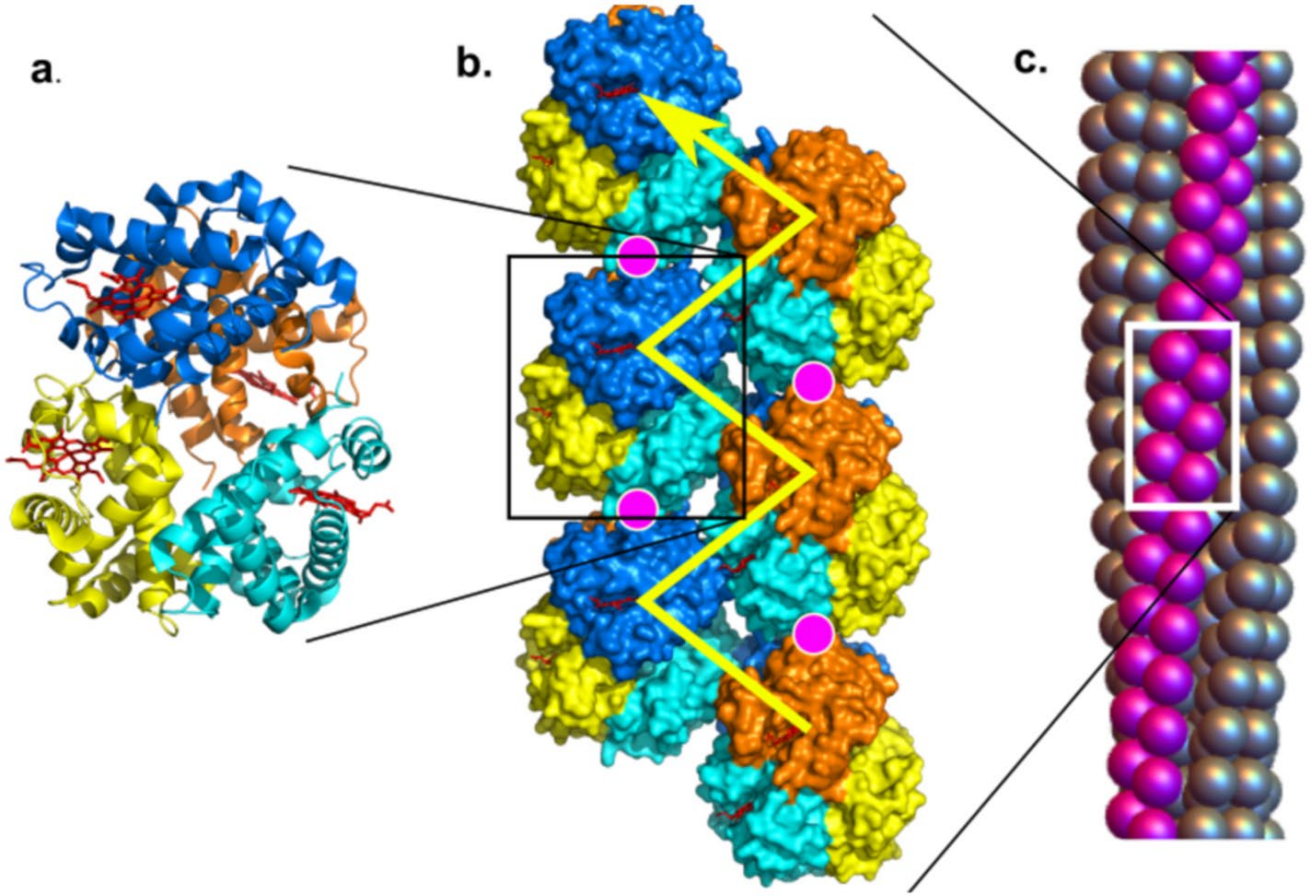
Polymer structure^32^. (**a**) The hemoglobin tetramer, containing 2 α chains (yellow and orange) and 2 β beta chains (cyan and blue). Tetramers form double strands (shown in panel (**b**)) that twist into polymers (panel **c**). In panel (**c**), each sphere represents a hemoglobin tetramer. The HbS β6V mutation docks into an adjacent β subunit pocket, forming lateral contacts, as shown by the yellow arrows. HbF lacks both donor and receptor; HbβT87Q lacks the donor and puts a bulky substitution in the receptor. α114 lies in an axial contact and is shown by the magenta circles. In a tetramer, residue 114 in either α chain packs against an adjacent molecule, so perturbing either inhibits effectively.

This inhibition follows from the stereochemistry of the fiber^11^. Sickle fibers comprise seven pairs of slightly-twisted, half-staggered strands of HbS molecules. The sickle-cell mutation (βE6V) lies at the diagonal contact point between two strands, denoted the lateral contact. In any given lateral contact, the mutant β6Val fits into an acceptor pocket on the β chain of an adjacent tetramer (yellow arrow in Fig. 2). Each β chain contains both a donor β6Val and an acceptor, and when one β chain on a given tetramer provides the donor, the other β chain on the same tetramer will employ its acceptor site. Thus, in a hybrid tetramer, replacing either β chain with a non-sickling variant inhibits fiber formation. The γ chains found in HbF suppress fiber formation because they can act as neither donor nor acceptor. In the construct βT87Q, designed to mimic the γ chain^12^, the threonine is replaced by a larger glutamine, reducing the size of the acceptor pocket and preventing insertion of the donor βV6.

The other principal contacts in the double strand are axial contacts, so named because they run along the fiber axis. This is where the Chiapas mutation is found (magenta circles in Fig. 2). Pro-114’s nearest neighbor across the axial contact is βE121, and the αP114R mutation introduces steric repulsion into this interface. Compensatory rotations of the tetramer can relieve this repulsion and allow formation of an Arg-Glu salt bridge, but weaken the lateral contact. Moreover, Pro114 on the other α chain in the tetramer forms an axial contact with αE116, and again replacing Pro with Arg generates repulsion. Hence, αP114R thwarts fiber assembly no matter which of the two α chains has been replaced in the Hb tetramer. We know of no other single amino acid substitution that inhibits in both positions.

Some hint of the inhibitory potential of HbChiapas has previously been seen^13^. The solubility of HbS-Chiapas was reported to exceed the input concentration of 33 g/dL. A 30% mixture of HbS-Chiapas with 70% HbS elevated the solubility to 21.3 g/dL (HbS solubility was measured as 16.3 g/dL). This factor of 1.3 increase in solubility is just what one gets by comparing the solubility of 30% HbF/S mixtures to the pure solubility of HbS, measured under slightly different conditions^3^. However, kinetic experiments carried out in high phosphate buffer were only briefly described in qualitative terms and no comparison was known for kinetics relative to HbF/S mixture.

Current strategies for expressing βT87Q via gene therapy produce roughly equal proportions of HbS and the mutant^6^. Using the data from Fig. 1 and the current models of the kinetics of polymer formation, we calculate that this level of expression will slow the homogeneous nucleation rate by a factor of 10^4^, and the exponential growth rate by a factor of 10^3^ (Fig. S1, Supplementary Information). While impressive, these numbers still leave room for improvement. For example, at present, 15% of the cells in patients treated with this gene therapy contain solely HbS^6^, and can therefore sickle and form occlusions during their 1-s passage through the microcirculation. When such cells occlude a capillary, they also immobilize the cells behind them, which are then depleted of O_2_ and become vulnerable to sickling themselves. If these trailing cells contain 50% HbF, our calculations indicate their sickling times would be around 30 s (Fig. S2, Supplementary Information), which may not be long enough to wait for an occlusion to resolve. Hence, even cells expressing the γ chain can sickle and accumulate the accompanying damage, ultimately leading to hemolysis.

In contrast, introducing a second αP114R gene should remove this risk. We anticipate that incorporating this mutant into the gene therapy vector would produce equal proportions of modified and native α chains, since the existing genes are not replaced^5,6^. This would lead to erythrocytes containing roughly 50% βT87Q and 50% Chiapas, which our calculations predict will not sickle at all on any physiologically relevant time scale (Fig. S2). In fact, the sensitivity of the kinetics to concentration amplifies these effects. For example, at 50% βT87Q, the overall rate of polymerization has slowed 100 times. Another 100-fold slowing only requires an additional 13% of non-polymerizing species (Fig. S2). Finally, we note that HbS Chiapas binds O_2_ with a normal binding curve^5^, avoiding any compromise of oxygen delivery in such a construct.

A number of other α chain mutants are known that inhibit fiber formation, including Hb Twin Peaks (L113H)^14^, Hb Stanleyville II (N78K)^15^, Hb Sealy (D47H)^16^, Hb Savaria (S49R)^17^, Hb J-Oxford (G15D)^16^ and Hb La Lamentin (H20G)^18^. A subtle but important feature of the Chiapas mutation site is that it interferes with polymer formation in either of the 2 possible arrangements. Different β chains participate as donor and acceptor regions in the fiber. Thus their α chain partners have two possible but distinct configurations in the polymer. Because the Chiapas mutation is located in the particular axial contact of the double strand, it acts as an inhibitor in either configuration. In contrast other α chain mutants act only in one configuration, and as such resemble the inhibition of HbA more than that of HbF as seen here. In particular it should be noted that the solubility of the above mutants are found in the range of 20–27 g/dL while the solubility of HbSChiapas was beyond 33 g/dL, and never measured. Thus, while it is true that other α chain mutants could be productively coupled with β chain gene therapy, the Chiapas mutation seems to be unique.

Our belief that improvement is possible is not to devalue the current therapy, which is changing the lives of patients. It is instead to point out that addition of this mutant to the repertoire of methods to prevent sickling represents a significant opportunity, particularly since it can be coupled with any of the gene-therapy strategies presently under study.

## Methods

We used the expression plasmid pHE2 to produce HbS Chiapas^19^. The plasmid was the generous gift of Prof. John Olson of Rice University and also included the sickle mutation at β6. The αP114R mutation was introduced using site-directed mutagenesis, using forward primer CATCTGCGTGCTGAATTTACCCCGGCTGTTCATGCGTCTCTGGATAAATTC and reverse primer GTAAATTCAGCACGCAGATGAGCAGCCAGAGTAACCAGCAGGCAGTGAG. The resulting plasmid was transformed into JM-109 cells and grown on LB-agar plates supplemented with 100 μg/mL ampicillin at 37 °C. A single colony was grown in 5 mL LB + ampicillin at 37 °C for several hours, which was then used to inoculate a 500 mL overnight culture in TB (1.2% tryptone, 2.4% yeast extract, 0.94% Potassium phosphate dibasic, 0.22% sodium phosphate monobasic, 100 μg/mL ampicillin) at 37 °C. 80 mL of the overnight culture was used to inoculate 1 L of TB-ampicillin supplemented with 2 mM magnesium sulfate and 1% glucose. The culture was shaken at 37 °C until the optical density reached 0.6–0.8, at which point 10 mg of bovine hemin (dissolved in freshly prepared 0.1 M NaOH) was added, along with IPTG to a final concentration of 0.2 mM. The flask was shaken for 20–24 h at 22 °C. The cells were harvested by centrifugation and cell pellets stored at − 80 °C.

**Figure 3:**
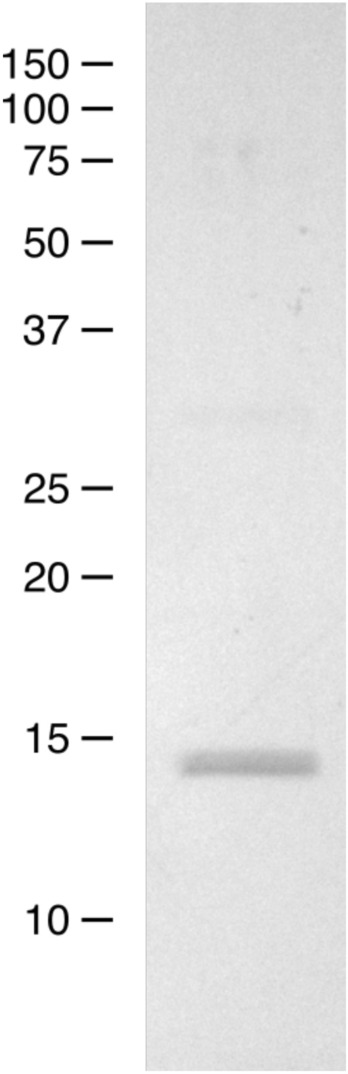
Coomassie-stained SDS-PAGE gel showing purified HbS Chiapas. Two closely spaced bands can be seen for the alpha and beta chains. The positions of molecular-weight markers are shown at left.

The cell pellets were thawed under running cold water and re-suspended in 40 mM Tris, pH 8.0, 1 mM benzamidine-HCl, 10 mM magnesium chloride, 1 mM manganese chloride, and 30 µg/mL DNase; 1 mg lysozyme was added per gram of cells. The mixture was stirred for 15 min at 4 °C, and then passed 3 × through an Avestin Emulsiflex C3 cell disrupter. The crude lysate was adjusted to pH 8.0 with Tris base and CO was bubbled through the mixture for 15 min. The material was spun for 45 min at 15,000×*g*, filtered through a 0.45-micron filter, and concentrated to 10–15 mL. The supernatant was applied to a 5-mL HiTrap Q HP column (Cytiva) equilibrated with 20 mM Tris, pH 7.4. The flow-through was dialyzed in 50 mM MDEA, pH 8.5 overnight at 4 °C. The following day, the protein was applied to a second 5-mL HiTrap Q HP column equilibrated in 50 mM MDEA, pH 8.5 and eluted with a gradient from 0 to 1 M NaCl. The resulting peak was dialyzed overnight in 20 mM sodium phosphate, pH 6.8 at 4 °C. The solution was then filtered and applied to a 5-mL HiTrap S HP column equilibrated in 20 mM sodium phosphate, pH 6.8; the column was washed and eluted using a pH gradient from pH 6.8 to 8.3. The protein was concentrated in an Amicon centrifugal concentrator, incubated with 10 mM sodium dithionate for 15 min, and exposed to CO gas for 15 min. Protein purity was assessed by SDS-PAGE (Fig. 3) (A fully detailed image may be found in SI). The correction of reversed heme orientations was carried out as previously described, and verified by circular dichroism^20^.

Sickle hemoglobin was purified by standard methods, including ion exchange chromatography^10^. Concentrations of both recombinant and unmodified HbS were measured with a novel procedure involving rectangular cross section capillaries with thicknesses that were verified by an interferometric device, and accounts for the reduced error bars in concentration in the new data. All samples were in 0.15 M Phosphate buffer, at pH 7.35, with 50 mM sodium dithionite. Data other than the HbS Chiapas recombinant mixture is taken from previously published works as indicated in the figure caption.

Continuous laser photolysis by a 2 W Coherent Verdi-G laser was used to probe the polymerization of sickle hemoglobin. The method, extensively described elsewhere^21^, uses a focused CW laser to continuously photolyze layers of sealed HbS solutions that were a few µm thick. The combination of focus and thinness allows heat to be dissipated efficiently. The CO that is photolyzed flows into solution, but upon extinction of the laser, CO will diffuse back and ultimately resaturate the illuminated area. This method is highly reversible and it has been shown that hundreds of exposures produce no discernible effects on the protein or its function^22^. Our experiments measure homogeneous nucleation by watching the variation of the onset of polymerization. That variation happens because the entire polymer mass is generated from a single nucleus, and the formation of a single nucleus is random by its nature.

Thus, the laser was incident on a steel mesh that yielded 59 spots of complete photolysis on the sample. Because the experiment involves the measurement of a distribution of polymerization-onset times, the multiplicity of spots allows for efficient data collection in lieu of successive exposures. To probe polymer formation, absorbance was used instead of light scattering. This works as a probe of polymer formation because as molecules are captured by the growing polymer arrays they can no longer efficiently diffuse out of the photolyzed volume, whereas, monomers outside the volume can diffuse inward, thus replenishing the monomers sequestered in immobile polymers^23,24^. This has the virtue that absorbance is demonstrably linear in polymerized hemoglobin concentration. The polymerization was monitored at 425 nm. This is close to an isosbestic in the deoxy-CO absorbance difference, so that the predominant contribution is simply due to increasing the mass of deoxyHbS in the given region, not converting COHb to deoxyHb (which is almost undetected at that wavelength). The small offset from the exact isosbestic permits helpful monitoring of where photolysis has occurred. The growth of absorbance (and thus mass) is exponential in time, and the value of the exponential is related to polymer mass according to an equation of the form

$$ \Delta  = A\;{\text{exp}}(Bt). $$

*B* is related to fundamental constants by *B*^2^ = *J*(*g* − d*f*/d*c*) where *g* is the heterogeneous nucleation rate and* J* is the polymer growth rate and *f* is the homogeneous nucleation rate^25^. Homogeneous nucleation is determined by analysis of a distribution that relates tenth-time probability to the overall nucleation rate. Szabo’s equation^26^ for the probability *T* of finding a given tenth time *t* is

$$ T(t) = \frac{{Be^{ - \zeta \langle t \rangle } }}{\Gamma (\zeta /B)}(1 - e^{ - Bt} )^{n} e^{ - \zeta t} $$

where *B* is as given above, Γ is a gamma function, *n* is a parameter related to the threshold of detection, $\langle t\rangle$ is the average tenth time in the absence of stochastics and $\zeta$ is the rate of homogeneous nucleation in the particular volume. $\zeta$ is related to the homogeneous nucleation rate constant *f* by the equation *f* = $\zeta$/*N*_*o*_*V* in which *N*_*o*_ is Avogardo’s constant, and *V* is the volume observed.

The solid curves in Fig. 1 have been drawn using theory we have previously developed and reported elsewhere^9,27^. The theory incorporates molecular crowding, which is significant, and does so from first principles so that no additional parameters are introduced^28^. Thus, the curves in which no copolymerization of hybrids is assumed uses the population of all species in calculating crowding, while only the population of the unhybridized HbS participates in the nucleus and its attendant thermodynamics. The fact that the mixture curves do not match the theory as well as the HbS data is likely due to the model needing unforeseen additions to encompass this behavior.

### Supplementary Information

Supplementary Information.

## Data Availability

The data is available on request from the corresponding author, Dr. F. A. Ferrone (fferrone@drexel.edu).
