# Supplementary material for: An α-chain modification rivals the effect of fetal hemoglobin in retarding the rate of sickle cell fiber formation
Source: Sci Rep. 2023 Dec 11;13:21997. doi: 10.1038/s41598-023-48919-3 (PMC10713580; doi:10.1038/s41598-023-48919-3)
Supplement: Supplementary file 1 — Supplementary Information. [file 41598_2023_48919_MOESM1_ESM.docx]

**Supplementary Information:**

**An α-chain modification rivals the effect of fetal hemoglobin**

**in retarding the rate of sickle cell fiber formation**

Eli H. Worth, Mark K. Fugate, Kimberly C. Grasty, Patrick J. Loll,

Marilyn F. Bishop and Frank A. Ferrone

**THEORY**

Because hemoglobin tetramers reversibly dissociate into αβ dimers, introducing any variant hemoglobin into a solution of sickle hemoglobin will lead to formation of hybrids; depending on the nature of the variant, these hybrids may or may not enter the HbS fibers. Even when the hybrids (and the non-HbS molecules) don't enter the fibers, their effects on polymerization extend beyond simple dilution of the HbS, because of the extreme concentrations of the Hb in the red cell. While the non-participating molecules don’t enter the fiber, they do crowd the solution, which offsets significantly the dilution factor, though it does not eliminate it. Such behavior is well described by the theory of molecular crowding (also called solution non-ideality). Here we outline the relevant theory and use it to predict how a solution of HbS will behave when non-polymerizing Hb is introduced.

Molecular crowding appears in this treatment in three distinct ways: As the monomer activity coefficients, as the activity coefficients of the homogeneous nucleus (taken as an approximately spherical entity), and as the activity coefficient of the heterogeneous nucleus, which is by definition attached to another polymer. An added subtlety is that the nuclei represent energetic turning points as a function of aggregate size, and therefore have sizes that are dependent on the solution conditions rather than being entities of fixed size.

The thermodynamic propensity to aggregate is given by the solubility and it can be expressed by equating the chemical potential of monomers in solution with that of monomers incorporated into polymers, as

*RT* ln γ_S_c_S_ = µ_PC_ + µ_PV_ – µ_RT_ (1)

_­_in which µ_PC_ and µ_PV_ are respectively the chemical potential for contacts and vibration of the center of mass of a molecule in the polymer, and µ_RT_ is the chemical potential of rotations and translations of a molecule in solution. *R* is the gas constant, and *T* is the absolute temperature.

Monomer activity coefficients γ are conveniently expressed as^1^

ln γ = 8*vc*­/(1–*vc*)^2^ (2)

where the specific volume of monomers is *v* and total monomer concentration is *c*.

Two principal equations describe polymer formation.^2^ The concentration of monomers that have been incorporated into polymers is denoted ∆ (defined as *c* – *c*_o_) and is given by

*d*Δ/*dt*  = *J cp* (3)

#### where c_p_ is the concentration of polymers, and *J* is the elongation rate.

*J = k_+_*γ*c – k­_-_ = k_+_(*γ*c –* γ­*_s_c_s_*) (4)

The concentration of polymers *c_p_*  then changes because of polymer creation by homogeneous nucleation, the rate of which is denoted by *f*, or by heterogeneous nucleation, which requires the existence of polymers, and therefore has a rate is proportional to the mass of polymers, ∆. Hence,

*dc*p/d*t* = *f + g*Δ (5)

These nonlinear equations have been solved for the initial growth phase by linearization and expansion around the start of fiber formation^3^, and thus the quantities of interest are subscripted by zero (*f_o_, g*_o_)to designate the initial value of a quantity that will change during the reaction.

#### Primary nucleation

The primary or homogeneous nucleation rate *f*o is the result of monomer addition to a spontaneously formed nucleus. If nuclei of size *i** have concentration denoted by ci* and possess activity coefficient γi* then the nucleation rate is given by^4^ ^2^;


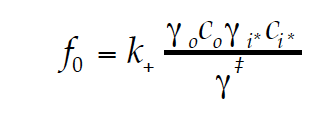


(6)

*k*+ is the monomer addition rate, taken as size independent. γ‡ is the activity coefficient for the activated complex, an aggregate of size i*+1. Because of the dissimilar size of nucleus and monomer, the description for γ from eqn 2 cannot be used for the nucleus. Taking both nucleus and monomer as roughly spherical allows the use of scaled particle theory to determine γ‡. ^2^


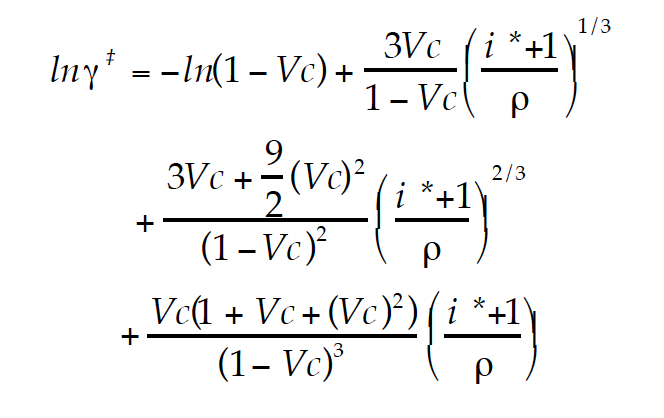


(7)

This depends on the nucleus size (*i**) and the concentration of hemoglobin *c*, but once given such input data γ‡ is fully specified with no adjustable parameters, viz.

Here *V* is the volume per millimole of monomer and *c* is the (total) monomer concentration. *i** is the nucleus size, which will be discussed below. The density of the nucleus relative to the monomer is given by ρ.

*ci** can be related to the contact energy, µ_­PC_ , and the chemical potential from vibrational entropy µ_PV_. For an aggregate of size *i*, the chemical potential of the aggregate depends on the fraction of contact sites in the infinite polymer that have been made, viz., δ(*i* ). The total contact energy for size *i* is given by

*µ*_iC_ ≡ *i* δ(*i* ) µ_PC_  ≈ (*i* + δ_1_ *ln i* + δ_2_ )µ_PC_ (8)

in which δ_1_ and δ_2_ are determined from fitting the above functional form of δ(*i*) to the contacts determined from close packed spheres ^5^. The total chemical potential from vibrations is

(*i –* 1) µ_PV_.

To simplify notation the parameter ξ is introduced, which contains μPC and other constants specified by the geometry of the nucleation process. ξ is defined by

ξ *= −*(4*+δ1μPC / RT)* (9)

In fitting nucleation rate data, µ_PC_ is determined by varying ξ while the geometrical parameters are unchanged. The homogeneous nucleation rate *f*o is given by


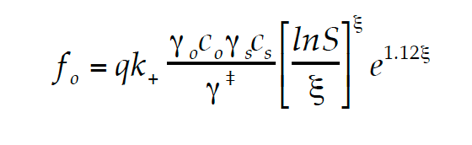


(12) (10)

where *q* contains only geometrically determined constants. (cf.^6^), and *S* is the activity supersaturation at the initial concentration co, defined as

*S* = γ_o_*c*_o_ */* γ*_s_c_s_* (11)

in which *c_s_* is the solubility, and γ*_s_* is the activity coefficient at solubility. c_o_ is the concentration of deoxyhemoglobin S at the initiation of the polymerization. γ_o_ is the activity coefficient of the total hemoglobin concentration at the start of polymerization. The nucleus size, *i**, is only used in the activity coefficient for the activated complex, eq 7. The expression for the nucleus size is

*i* =* ξ */ lnS.* (12)

Crowding effects appear in the activity coefficient for the monomer, γ, which depends on the total concentration of hemoglobin and in γ‡, the activity coefficient for the activated complex, an aggregate of size *i**+1. This depends on the nucleus size (*i**) and the concentration of hemoglobin *c*, but since *i** is determined without added parameters or variables, γ‡ is fully specified. ξ does not change as non-polymerizing species are added.

#### Secondary Nucleation

The rate of secondary or heterogeneous nucleation *g_o_*∆ is proportional to the concentration of monomers already present in polymers ∆. Analogous to equation 10,


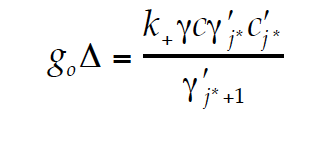


(13)

Since aggregates of size *j** are attached to a polymer here, we denote their concentration and activity coefficients with a prime. The activity coefficient of the attached aggregate includes the volume excluded by the polymer to which the aggregate is attached. The activity coefficient in the denominator is that of the activated complex, an attached aggregate of size *j**+1, which again includes the polymer in the calculation. Since the heterogeneous nucleus consists of an aggregate attached to a polymer, the activated complex is no longer is a spherical object. The heterogeneous nucleus, an attached aggregate, is written with a prime to distinguish it from a free aggregate like the homogeneous nucleus, which would merely differ in size.

The activity of an attached aggregate of size *j** to polymers is given by its equilibrium with the solution aggregates and the polymer sites to which it attaches, i.e.,

γ*′j** *c′_j_* = K j′* γj* cj* γ*p φ Δ (14)

in which primes indicate attached aggregates, and unprimed symbols indicate solution aggregates. *K*’_j*_ is the equilibrium constant for the attachment process, and ∆ is the concentration of monomers in polymers. φ is a constant described below. Then equation 14 becomes


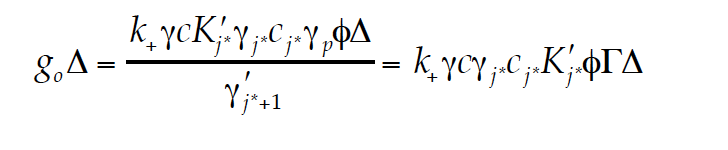


(15)

in which Γ is here defined as γ_p_/γ’_j*+1_, *i. e.,*  the activity coefficient for a polymer with no aggregate attached divided by the activity coefficient of a polymer with aggregate size j*+1 attached . Γ, originally thought to be unity, is approximated by^7^


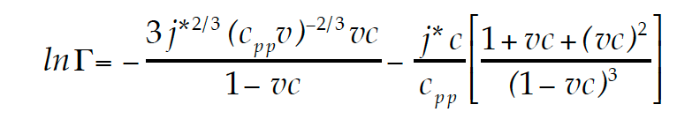


(16)

where *c­_pp_* is the concentration of hemoglobin in the polymer phase, and *v* is the specific volume of the monomer as also used in eq 7. The size of the heterogeneous nucleus *j** is computed in a thermodynamic treatment similar to that used for the homogeneous nucleus, but with the addition of an energy of attachment. The additional terms that appear in the calculation of the attachment can be traced to *K*’_j*_φ in eqn. 15. In energetic terms the added stability of the heterogeneous nucleus is

*– RT* ln *K’*_j*_ φ = – *RT* ln φ + µ_CC_ σ_1_ *j** + µ_CC_ σ_2_ ln *j**  (17)

µ_CC_ is the chemical potential per contact area between polymers and thus is the energetic term causing a heterogeneous nucleus to stick to the polymer. In contrast, µ_PC_ is the energetic term describing the contact energy of a monomer within the polymer. The surface area in contact has linear and logarithmic contributions with coefficients σ_1_ and σ­­_2_ . This is similar to the expansion of the contact energy within the homogeneous nucleus in constant, linear and log terms (recall eqn. 10). Originally φ was taken to specify the fraction of surface molecules available. Physically it is impossible from kinetic measurements to distinguish between a small number of sites (small φ) and a larger number of sites with weaker attachment energies.

_
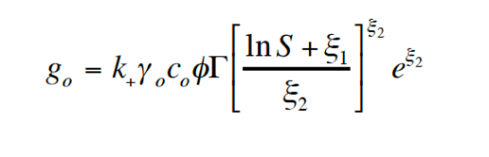
_From above definitions, analogous to eqn 10,

_­_

(18a)

where

ξ*1= −σ1 μCC / RT* (18b)

and

ξ2 = ξ*+* 4 *+ σ2 μCC / RT* (18c)


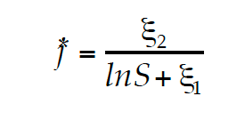
*S* is the activity supersaturation as above. ξ is also the same variable used in eqn 9. In this notation the heterogeneous nucleus size *j** can be written as

(19)

The heterogeneous nucleus size *j** does not depend on φ.

**Observables**

The above description describes the fundamental rates that give rise to sickle fibers: homogeneous nucleation in solution followed by secondary (heterogeneous) nucleation on other polymers, both of which create growing fibers that consume monomers from a solution or cell. To determine these fundamental rates, two observations are customarily made.

The solution to the set of differential equation 3 and 5 is given under most circumstances by

∆ = (*A*/2) (exp (*Bt*) – 1) (20)

The constants *A* and *B* are related to the other parameters by

*B*^2^ = *J* (g_o_ – *df_o_*/*dc*) (21)

while

*A* = *f_o_/* (g_o_ – *df_o_*/*dc*) (22)

from which it is apparent that

*B^2^A = Jf_o_*  (23)

This exponential growth gives an appearance of a delay, which is a designation often used in more qualitative descriptions. This delay time can be made more rigorous by defining the tenth time, *t*_1/10_ as the requisite time for ∆ to reach (*c_o_* – *c*_s_*)/*10. (cf inset to Fig S1) It is then possible to show that

*t*_1/10_ = ln [(*c_o_* – *c*_s_)/5*A +*1] / *B.*  (24)

One observable is therefore the tenth time, which is useful in many ways for relating time when a significant polymer mass begins to form. Since polymer growth is exponential, it is straightforward to fit the polymerization mass ∆ with an exponential function and obtain the parameter *B.*

When polymerization occurs within a sufficiently small volume, the volume can be filled with polymers following one primary nucleation event before a second nucleation event occurs. This is easier than it might first seem, because nucleation is so concentration-dependent that consumption of monomers quickly shuts down that process relative to the less-concentration-sensitive growth of fibers. Since the first nucleation event will be intrinsically random (as the formation of a nucleus is inherently a random process) the entire polymerization event will commence in random fashion. This distribution can be related to fundamental rates, and therefore the distribution of starting times can be analyzed so as to reveal the primary nucleation rate. The stochastic distribution *T*(*t*) of the tenth times follows the equation^8^:


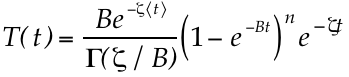


(25)

where *B* is as given in eq (20-21), and Γ in this equation is a gamma function of the argument ζ/*B* , *n* is a parameter related to the threshold of detection, ⟨ *t* ⟩ is the average tenth time in the absence of stochastics and ζ is the rate of homogeneous nucleation in the particular volume. ζ is related to the homogeneous nucleation rate constant *f_o_* by the relationship

*f_o_ =* ζ /*N_o_V*_o_ (26)

in which *N_o_* is Avogadro’s constant, and *V_o_* is the volume observed.

In the work reported here, we measured the primary nucleation rate by the stochastic method described, and measured *B* by observing exponential growth. These observables vary as the fraction of polymerizable species is changed for a given total concentration. This is illustrated in Fig. S1 for a concentration of 34 g/dl. It is especially notable how the effects are magnified as the fraction of non-polymerizable species rises.

We could then use these constants to deduce tenth times for simple comparison with expectation. Figure S2 shows the expected tenth times for a 34 g/dl sample containing HbS and a non-HbS component that does not participate in the polymer, either as tetramers or as hybrids. Once the fraction of the non-polymerizable component reaches 50%, the tenth time has been extended to ca. 10 s, well beyond the time it would take a cell to transit a capillary (note that the tenth time is measured from the point of complete deoxygenation; as we have discussed elsewhere^9^, the presence of oxygen will substantially delay time to sickling).

For mixtures containing a fraction X_N_ of non-polymerizing β chains, such as βT87Q, or γ chains, binomial statistics give the following fractions: (1-X_N_)^2^ species that are pure HbS, 2 X_N_ (1-X_N_ ) species that are hybrids, and also don't polymerize, and (1-X_N_)^2^ that are pure non-S. If hybrids do not polymerize, then a solution containing 50% of such chains possesses 25% of the sample capable of fiber formation. When these combine with a fraction X_C_  of Chiapas, the terms


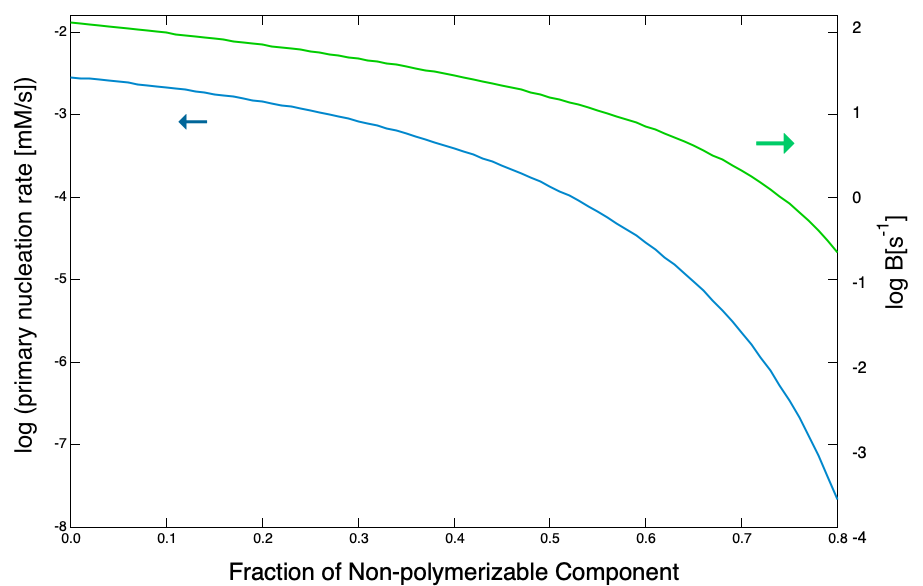


**Figure S1.**  Log of primary nucleation rate and log of exponential growth parameter B as a function of the fraction of non-polymerizable component. The calculations use the equations in the text for a 34 g/dl solution at 37°C following complete deoxygenation. The non-polymerizable component is assumed to not participate in the fibers as a tetramer nor as hybrids. The left axis refers to primary nucleation rate, the right axis refers to B. The strong dependence is due to the sensitivity of nucleated polymerization.

describing the distribution of β chains are now multiplied by corresponding binomially derived terms for the α chains, resulting in the distributions shown in Table S1, below. When X_N_= X_C_=0.5, the net fiber-forming component is 0.5^4^ = 0.0625, and the remaining 94% of the sample will not be able to enter fibers, due to the presence of some combination of fiber-inhibitory subunits. This would make the kinetics impossibly long, so that no polymerization will occur on any physiologically relevant time scale.

**Figure S2.**  Log tenth time as a function the fraction of non-polymerizable component. The calculation, following the equations in the text, is for a 34 g/dl solution following complete deoxygenation. The non-polymerizable component is assumed to not participate in the fibers as a tetramer nor as hybrids. The inset illustrates the definition of the tenth time, viz. the time, as denoted by the red dot, required for polymerization to reach 1/10 of its final value. Blood cells typically require around one second to transit capillaries.

**Table S1**

Frequency of Occurrence of Various Mixture Species

| Species* | Statistical Probability Equation | Probability when  X_C_ = X_N_ = 0.5 | Fiber participation? |
| --- | --- | --- | --- |
| α_2_β^S^_2_ | (1-X_C_)^2^(1- X_N_)^2^ | 0.0625 | 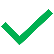 |
| α_2_β^N^β^S^ | 2(1-X_C_)^2^(1-X_N_)X_N_ | 0.125 | **X** |
| α_2_β^N^_2_ | (1-X_C_ )^2^X_N_^2^ | 0.0625 | **X** |
| αα^C^β^S^_2_ | 2X_C_(1-X_C_)(1- X_N_)^2^ | 0.125 | **X** |
| αα^C^β^N^β^S^ | 4X_C_(1-X_C_)(1-X_N_)X_N_ | 0.250 | **X** |
| αα^C^β^N^_2_ | 2X_C_(1-X_C_)X_N_^2^ | 0.125 | **X** |
| α^C^_2_β^S^_2_ | X_C_^2^(1- X_N_)^2^ | 0.0625 | **X** |
| α^C^_2_β^N^β^S^ | 2X_C_^2^(1-X_N_)X_N_ | 0.125 | **X** |
| α^C^_2_β^N^_2_ | X_C_^2^X_N_^2^ | 0.0625 | **X** |

*****β^S^ is a sickle β chain, β^N^ is a chain that cannot polymerize, such as βT87Q, α^C^ is a Chiapas α chain.

**SAMPLE PURIFICATION**

The entire Coomassie-stained gel corresponding to the image shown in Figure 3 is presented below. The leftmost two lanes contain molecular-weight markers, with sizes indicated; the next eight lanes contain increasing amounts of purified HbS Chiapas. The lane shown in Figure 3 is indicated here with an arrow. The impurity having a molecular weight of approximately 23 kDa is likely the endogenous *E. coli* SodA protein (superoxide dismutase), as indicated by mass spectrometric data (not shown); we hypothesize that the *sodA* gene is overexpressed in response to oxidative stress associated with high levels of heme.

**Figure S3**: Entire Coomassie-stained gel corresponding to the image shown in Figure 3, described above.

**REFERENCES**

1. Ferrone, F.A. & Rotter, M.A. *J Mol Recognit* **17**, 497-504 (2004).

2. Ferrone, F.A., Hofrichter, J. & Eaton, W.A.*J. Mol. Biol.* **183**, 611-631 (1985).

3. Bishop, M.F. & Ferrone, F.A. *Biophys. J.* **46**, 631-644 (1984).

4. Hill, T.L. *An Introduction to Statistical Thermodynamics*, (Dover Publications, New York, 1986).

5. Ginnel, R. *J. Chem. Phys.* **34**, 992-998 (1961).

6. Ivanova, M., Jasuja, R., Kwong, S., Briehl, R.W. & Ferrone, F.A. *Biophys. J.* **79**, 1016-1022 (2000).

7. Ferrone, F.A., Ivanova, M. & Jasuja, R. *Biophys. J.* **82**, 399-406 (2002).

8. Szabo, A. *J. Mol. Biol.* **199**, 539-542 (1988).

9. Yosmanovich, D., Rotter, M., Aprelev, A. & Ferrone, F.A. *J Mol Biol* **428**, 1506-1514 (2016).
